# Chitosan Silver and Gold Nanoparticle Formation Using Endophytic Fungi as Powerful Antimicrobial and Anti-Biofilm Potentialities

**DOI:** 10.3390/antibiotics11050668

**Published:** 2022-05-16

**Authors:** Ehab M. Mostafa, Mohamed A. Abdelgawad, Arafa Musa, Nasser Hadal Alotaibi, Mohammed H. Elkomy, Mohammed M. Ghoneim, Mona Shaban E. M. Badawy, Mostafa N. Taha, Hossam M. Hassan, Ahmed A. Hamed

**Affiliations:** 1 Department of Pharmacognosy, College of Pharmacy, Jouf University, Sakaka 72341, Saudi Arabia; emmoustafa@ju.edu.sa (E.M.M.); akmusa@ju.edu.sa (A.M.); 2 Department of Pharmaceutical Chemistry, College of Pharmacy, Jouf University, Sakaka 72341, Saudi Arabia; 3 Department of Clinical Pharmacy, College of Pharmacy, Jouf University, Sakaka 72341, Saudi Arabia; nhalotaibi@ju.edu.sa; 4 Department of Pharmaceutics, College of Pharmacy, Jouf University, Sakaka 72341, Saudi Arabia; mhalkomy@ju.edu.sa; 5 Department of Pharmacy Practice, College of Pharmacy, AlMaarefa University, Ad Diriyah 13713, Saudi Arabia; mghoneim@mcst.edu.sa; 6 Pharmacognosy and Medicinal Plants Department, Faculty of Pharmacy (Boys), Al-Azhar University, Cairo 11884, Egypt; 7 Department of Microbiology and Immunology, Faculty of Pharmacy (Girls), Al-Azhar University, Cairo 11884, Egypt; mony.badawe@yahoo.com; 8 Department of Microbiology and Immunology, Faculty of Pharmacy, Nahda University, Beni-Suef 62764, Egypt; moustafa.nasr@nub.edu.eg; 9 Department of Pharmacognosy, Faculty of Pharmacy, Beni-Suef University, Beni-Suef 62513, Egypt; 10 National Research Centre, Microbial Chemistry Department, 33 El-Buhouth Street, Dokki, Giza 12622, Egypt; ahmedshalbio@gmail.com

**Keywords:** chitosan–nanometal conjugate, endophytic fungi, antimicrobial, anti-biofilm

## Abstract

Nanotechnology is emerging as a new technology with encouraging innovations. Global antibiotic use has grown enormously, with antibiotic resistance increasing by about 80 percent. In view of this alarming situation, intensive research has been carried out into biogenic nanoparticles and their antibacterial, antifungal, and antitumor activities. Many methods are available to enhance stability and dispersion via peroration of conjugate with a polymer, such as chitosan, and other bioactive natural products. Two marine fungi were isolated and identified as *Aspergillus* sp. and *Alternaria* sp. via sequencing of the 16S rRNA gene. In this work, these strains were used to form the conjugation of biogenic silver nanoparticles (AgNPs) from *Aspergillus* sp. Silv2 extract and gold nanoparticles (AuNPs) from *Alternaria* sp. Gol2 extracts with chitosan to prepare chitosan–AgNPs and chitosan–AuNP conjugates. A variety of imaging and analytical methods, such as UV–vis, X-ray powder diffraction (XRD), FTIR spectroscopy, transmission electron microscopy (TEM), and scanning electron microscopy (SEM) were utilized to characterize biogenic nanoparticles and conjugates. The biosynthesized Ag and Au nanoparticles along with the prepared conjugates were evaluated for their antimicrobial effects on Gram-negative and Gram-positive bacterial isolates, including *Escherichia coli* and *Staphylococcus aureus*. Both chitosan–AgNP and AuNP showed powerful antimicrobial activities compared to the control. On the other hand, chitosan–AgNP conjugation had better antibacterial ctivity than chitosan–AuNPs, which exhibited moderate activity against *S. aureus* and very low activity against *E. coli*. Furthermore, the antibiofilm potentials of the prepared conjugates were tested against four biofilm-forming bacteria, including *P. aeruginosa*, *B. subtilis*, *E. coli*, and *S. aureus*. The obtained results indicate that the chitosan–AgNP showed a promising anti-biofilm activities on all strains, especially *S. aureus*, while chitosan–AuNP conjugates showed moderate anti-biofilm against *B. subtilis* and weak activities against the other three strains. These results showed the superiority of chitosan–AgNP as a promising antibacterial as well as biofilm formation inhibitors.

## 1. Introduction

The continuous increase in antimicrobial resistance (AMR) is one of the world’s most serious health issues. It happens when bacteria, viruses, fungi, and parasites mutate and stop responding to chemotherapeutic agents [1]. As a result, certain harmful bacteria have developed resistance to a variety of antibiotics, presenting challenges in the development of novel antibacterial compounds that can regulate bacterial resistance. The antibacterial properties of silver and silver-based ions have long been known [2,3]. Several elements have been shown to have an important role in the persistence of bacteria, including gene differential expression, extracellular matrix secretion, trapping of antibiotic-degrading enzyme in high concentrations, and biofilm formation, according to recent discoveries. One of these important factors is the formation of bacterial biofilm, which facilitates the colonization of microorganisms on living and non-living surfaces. Some reports have declared that 65% to 80% of all clinical infections are caused by pathogenic bacteria such as *E. coli* and *S. aureus* that have the ability to form biofilm [4]. In addition, this adaptive phenomenon makes bacteria between 10 and 1000 times more resistant to common antibiotics [4].

Nanoparticles have attracted much attention from researchers. These nanobiotechnology applications are useful in various fields, such as in medicine; for industrial devices, such as biosensors; and in catalytic, therapeutic, and antibacterial materials [5].

Among all metal nanoparticles, silver nanoparticles have attracted significant interest due to their unique physical and biological characteristics, such as conductivity, chemical stability, and catalytic capabilities [6]. Besides these properties, silver nanoparticles have anti-viral, antibacterial, antifungal, and anti-inflammatory properties that can be used in composite fibers, cosmetics, cryogenic superconducting materials, electrical components, and the food sector [6].

In biomedical applications, the silver nanoparticle has been utilized recently in wound dressings, antiseptic sprays, topical creams, and fabrics. On the other hand, silver nanoparticles also function as antiseptic agents, with broad biocidal activity against pathogenic bacteria via accumulation on the cell membrane, which disrupts its function [5].

Microbiologically manufactured nanoparticles are shown to be more favorable for use than their chemically synthesized counterparts, since the former do not demand the same rigorous conditions as the latter, such as purity of the starting material. Additionally, microbiologically produced nanoparticles (NPs) are more economically feasible, as their production involves ambient conditions and relatively cool temperatures (20–30 °C) [7]. Furthermore, the inclusion of a biological capping agent on certain micro-biogenic nanoparticles, which acts as a protective coating against oxidation, agglomeration, and aggregation, results in increased stability. As a result, microbiologically produced NPs are frequently regarded as a superior antibacterial treatment alternative [7].

Biological synthesis of AgNPs can be achieved using a reducing agent. Microorganisms such as fungus, bacteria, actinomycetes, or algae can be used for this purpose. These microbes, with their capacity to synthesize many proteins, have been linked to metal ion reduction and nanoparticle shape control [8,9]. However, the production of AgNPs from fungi can be carried out either extracellularly or intracellularly [8].

In many aspects, fungi are superior to other microbes. They are simpler to hold and control, even if they take more precision and care to develop. They produce vast quantities of enzymes. Biogenically prepared polymeric nanoparticles have recently gained tremendous attention, due to their superior bioactivity, including antioxidant and antibacterial actions, against antibiotic-resistant microorganisms [10]. Chitosan (CS) is a linear polysaccharide made up of (1-4)-D-glucosamine and N-acetyl-D-glucosamine, and is produced from the exoskeletons of crustaceans and insects via a deacetylation process [11]. The biocompatibility, degradability, nontoxicity, and permeability of CS are all properties with advantageous potential in the medical field [12]. Antimicrobial use [13,14], wound healing [15,16], drug carrier use [17,18], biosensor use [17], and water purification [19] are only a few of the biomedical uses of CS. To optimize its activity, CS needs suitable structure modification, including size and shape modification. This modification can be achieved by converting the normal CS structure to nanoparticle form (1–1000 nm), utilizing chemical, mechanical, or biological techniques. Smaller chitosan nanoparticles are produced using the biological method. The goal of this research was to synthesize AgNPs and AuNP nanoparticles using marine fungi from various habitats. In addition, this study aimed to conjugate green synthesized AgNPs and Au chitosan to prepare chitosan–AgNPs and –AuNP conjugates. Furthermore, this research investigated the antimicrobial and antibiofilm effects of the green synthesized AgNPs, chitosan-NPs, and chitosan–AgNP conjugates.

## 2. Materials and Methods

### 2.1. Soil Sample Collection and Fungus Isolation

Soil samples were collected from Zagazig, Egypt, during September 2019. The collected samples were coded and stored in a cooled sterilized container until they reached the lab. The samples were processed in an isolation process using the soil dilution method [18] and inoculated on potato dextrose agar plates [19] for 10 days at 25 °C until the fungal colonies appeared. To inhibit any bacterial and fungal growth, 30 mg L^−1^ each of streptomycin and rose bengal were added separately to the medium culture. The separated fungal colonies were preserved on potato dextrose agar slants.

### 2.2. Genetic Identification of Fungal Strains

The two fungal strains were isolated and cultured in potato dextrose broth media for 5 days at 25 °C. Pure separate colonies were spread in sterile saline solution (0.5 mL). The suspension was then centrifuged at 10,000 rpm for 10 min/RT. The extraction of DNA was carried out with the help of DNeasy Blood & Tissue Kits according to the manufacturer’s instructions. Two primers were used: ITS2, GCTGCGTTCTTCATCGATGC and ITS3, GCATCGATGAAGAACGCAGC. The amplification reaction mixture was then prepared: 20 µL of PCR reaction solution were combined with 1.0 µL of DNA solution, and PCR reaction profile was as follows: denaturation for 5 min at 94 °C, followed by 35 cycles of 30 s at 94 °C, then 30 s at 55 °C, 90 s at 72 °C, and a final extension step of 5 min at 72 °C. A Montage PCR Clean-up kit (Millipore) was used to remove unincorporated PCR primers and dNTPs from PCR products. Sequencing of the purified PCR product was carried out via two primers: ITS1, TCCGTAGGTGAACCTGCGG and ITS4, TCCTCCGCTTATTGATATGC. The sequencing findings were resolved using an Applied Biosystems model 3730-XL automated DNA-sequencing machine.

### 2.3. Cultivation of Fungi and Preparation of Culture Filtrate

The potato broth medium was used for the cultivation of the two isolated fungal strains. Each strain was incubated in 250 mL broth medium for 9 days. Mycelia were removed by centrifugation at 5000 rpm for 30 min, while the mycelium-free supernatants were used for the biosynthesis of silver and gold nanoparticles.

### 2.4. Biosynthesis of Gold and Silver Nanoparticles

Twenty-five milliliters of mycelium-free supernatant for each fungus (*Aspergillus* sp. and *Alternaria* sp.) was incubated with 25 mL of silver nitrate and HAuCl4 solution 10^−3^ mol L^−1^, and incubated at 25 °C 150 rpm for 72 h. The mixture’s hue changes were an indication of silver ion reduction, forming silver and gold nanoparticles.

### 2.5. UV–Vis Spectral Analysis

The biogenesis of AgNPs and AuNPs was first seen as a change in the hue of the solution. The transformation was tracked by taking aliquots (1 mL) of the mixture and monitoring the UV–vis spectra of the solutions using a SPECTROstar nano absorbance plate reader (BMG LABTECH GmbH, Allmendgrun, Germany).

### 2.6. Studies of X-ray Diffraction (XRD)

The X-ray diffraction (XRD) pattern of the generated green AgNPs was measured using a PAN analytical X’pert PRO X-ray diffractometer (Philips, Eindhoven, Netherlands), with Cu Ka1 radiation and operating voltage and tube current of around 40 kV and 30 mA. After the material was drop-coated onto a glass substrate, the X-ray diffraction patterns were collected at 2θ from 10° to 80° at a scanning speed of 0.02°/min.

### 2.7. Fourier-Transform Infrared Spectroscopy (FTIR)

Silver nanoparticle spectra were obtained using the Broker vertex 80 v in the range 4000–400 cm^−1^ with a resolution of 4 cm^−1^, in accordance with Brock-Neely (1957).

### 2.8. Transmission Electron Microscopy Analysis (TEM)

Transmission electron microscopy (TEM) was used to evaluate the size and form of the created silver nanoparticles. For sample preparation, measures of 2–4 μL of silver or gold nanoparticle solution were dispensed onto carbon-coated copper grids. The thin films were air-dried and detected using a Philips 10 Technai with a wavelength (λ) of 0.0251 and an accelerating voltage of around 180 keV.

### 2.9. Preparation of Chitosan–AgNP and Chitosan–AuNP conjugates

The synthesis of chitosan–AgNP and chitosan–AuNP conjugates was carried out as described by Bilal et al., 2019. Briefly, a chitosan solution (0.5% *w*/*v*), (commercial grade, Med DDA%—Low-Medium Mw with Degree of Deacetylation (DDA)—88.3% Viscosity (mpa·s, 20 °C)—70) was ultrasonically dispersed and dropped into 5.0% (*w*/*v*) acetic acid solution over 1 h at 28 ± 2 °C with stirring. Biogenic AgNPs and AuNPs were added in a concentration of 10% (*w*/*v*), drop by drop; each was extruded into a 20 mL chitosan solution and swirled continuously at 120 rpm. Before being poured into a sterile, labeled Petri plate and incubated in a hot air oven at 50 °C for 24 h to develop chitosan–AgNP and –AuNP conjugates, the above mixture was activated for another 2.0 h with 0.5% (*w*/*v*) glutaraldehyde solution (freshly prepared in a 50 mM Na-malonate buffer at pH 4.5).

### 2.10. Antimicrobial Assay

The antibacterial activity of the nanoparticles was tested in 96-well flat polystyrene plates using one Gram-negative bacteria (*E. coli* ATCC 25955) and one Gram-positive bacteria (*S. aureus* NRRL B-767). The plates were incubated at 37 °C overnight with 10 µL of test extracts (final concentration of 50 g/mL) added to 80 µL of lysogeny broth (LB broth) and 10 µL of bacterial culture suspension (log phase). After incubation, good antibacterial activity in the tested drug was indicated by visible clearing in the wells, while chemicals that did not affect the bacteria caused the growth media in the wells to appear opaque. In a Spectrostar Nano Microplate Reader, the absorbance was measured after roughly 20 h at OD600 (BMG LABTECH GmbH, Allmendgrun, Germany).

### 2.11. Antibiofilm Assay

Ninety-six-well flat polystyrene plates were used to investigate the biofilm-inhibitory efficacy of the extracts against four clinical microorganisms, including Gram-positive bacteria (*S. aureus* and *B. subtilis*) and Gram-negative bacteria (*P. aeruginosa* and *E. coli*). Briefly, each well was filled with 180 µL of lysogeny broth (LB broth), then seeded with 10 µL of pathogenic bacteria, followed by 10 µL of samples and control (final concentration of 500 µg mL^−1^) (without test sample). The plates were incubated for 24 h at 37 °C, following which the contents of the wells were removed and washed in 200 µL of phosphate buffer saline (PBS) pH 7.2 to eliminate any free-floating bacteria, and then dried in sterilized laminar flow for 1 h. For staining, 200 µL of crystal violet (0.1 percent *w*/*v*) was applied to each well for 1 h, then the excess stain was removed and the plates were left to dry. After cleaning the dried plates with 95% ethanol, the optical density was measured using a Spectrostar Nano Microplate Reader at 570 nm. (BMG LABTECH GmbH, Allmendgrun, Germany).

## 3. Results and Discussion

### 3.1. Isolation of the Fungal Isolate from Different Marine Samples

Two fungal strains were isolated from the collected soil sample using the serial dilution method. The fungal colonies were distinguished based on their morphological characteristics. The obtained strains were tagged and stored at 4 °C in a culture collection at Egypt’s National Research Center Microbial Chemistry Department.

### 3.2. Genetic Identification of the Isolated Fungal Strains

**Figure 1:**
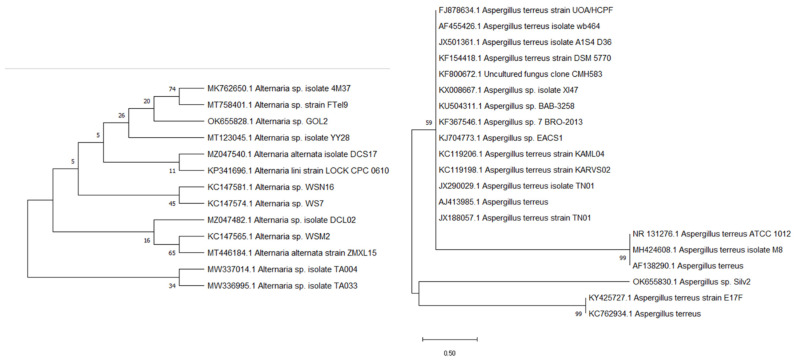
Evolutionary trees of the *Aspergillus* sp. Silv2 and *Alternaria* sp. Gol2.

Based on the preliminary evaluation of the isolated fungi, the fungal strain was identified by sequencing of the 16S rRNA gene. The DNA from the two isolates was extracted, identified, and matched against other known sequences in the GeneBank database using the BLAST program to determine the similarity score and statistical significance of the matches (http://www.blast.ncbi.nlm.nih.gov/Blast, accessed on 1 January 2022). The results revealed that the 18S rRNA gene sequences of the isolate Silv2 and the isolate Gol2 were similar, with the Silv2 having 100% similarity with *Aspergillus* sp. and the Gol2 having 100% homology with *Alternaria* sp. The evolution history (Figure 1) was calculated using the Maximum Likelihood technique and the Tamura–Nei model [20]. The proportion of trees in which the related taxa are grouped is shown next to the branches. The Tamura–Nei model was used to generate a matrix of pairwise distances, and the topology with the highest log-likelihood value was chosen as the beginning tree for the heuristic search. MEGA X was used to conduct the evolutionary research [21].

### 3.3. Biogenic Synthesis of Silver and Gold Nanoparticles

**Figure 2:**
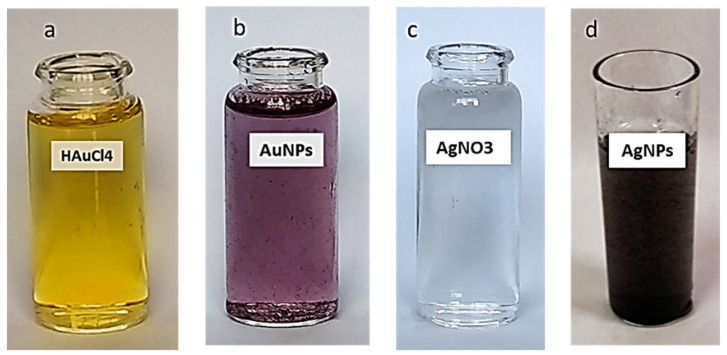
Change in color due to the formation of nanoparticles. (**a**) HAuCL4, (**b**) AuNPs, (**c**) AgNO3, and (**d**) AgNPs.

The formation of AgNPs and AuNPs was clearly visible in the color change upon the addition of the *Aspergillus* sp. Silv2 supernatant to the AgNO3 aqueous solution and of the *Alternaria* sp. Gol2 supernatant to the HAuCl4 aqueous solution. The formation of silver nanoparticles (AgNPs) and gold nanoparticles (AuNPs) was mediated at 30 °C under dark conditions after the addition of fungal sp. supernatants. The supernatant became yellowish-brown after interacting with Ag+ ions, indicating metal ion reduction and the formation of silver nanoparticles. Due to the activation of surface plasmon oscillations in the particles, UV–vis absorption spectroscopy indicated the formation of AgNPs with the formation of pale yellow to brown color (Figure 2d). Additionally, the shift in the color of a gold mixture from yellow to purple confirmed the formation of AuNPs with the addition of *Alternaria* sp. Gol2 supernatant. (Figure 2b)

### 3.4. Characterization of Biosynthesized AgNPs and AuNPs

**Figure 3:**
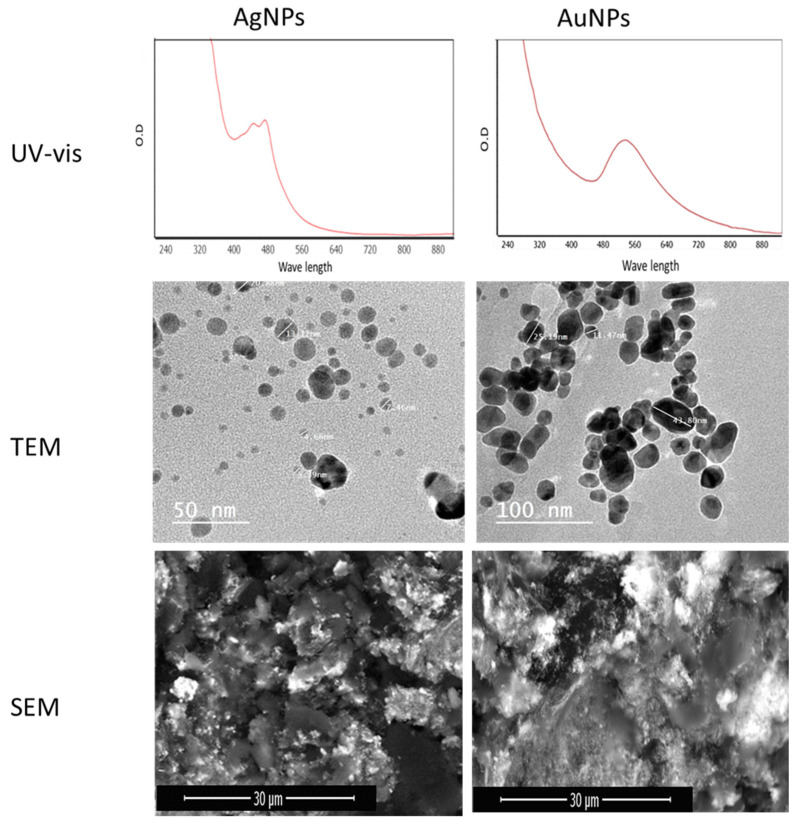
UV–vis, TEM, and SEM micrographs for biosynthesized AgNPs and AuNPs.

AgNPs were generated in the presence of *Aspergillus* sp. in Silv2 supernatants. The surface plasmon resonance (SPR) absorption spectra ranged from 400 to 550 nm, indicating that AgNPs were generated at different times. The surface plasmon band (SPR) of the produced AuNPs was at 550 nm, while the surface plasmon resonance (SPR) absorption spectral range was from 450 to 650 nm (Figure 3). Transmission electron microscopy (TEM) and field emission scanning electron microscopy (FESEM) were used to investigate the size and morphology of the green AuNPs and AuNP particles. The AgNPs had an average particle size of approximately 4.5 ± 20 to 50.2 ± 74 nm with a sphere-like shape, according to the TEM micrograph (Figure 3). Transmission electron microscopy (TEM) and field emission scanning electron microscopy (FESEM) were utilized to observe the particle size and form of the AuNPs (Figure 3). The AuNPs had an average particle size of ∼3.47 ± 2 to 35.50 ± 2 nm with spherical and polydisperse AuNPs (Figure 3).

### 3.5. Preparation of Chitosan–AgNP and –AuNP Conjugates

The chitosan–AgNP and –AuNP conjugates were prepared using biosynthesized *Aspergillus* sp. AgNPs and *Alternaria* sp. AuNPs. Chitosan derivatives and chitosan-nanoparticles (NP) generally have a positive surface charge and mucoadhesive characteristics, allowing them to stick to mucus membranes and release the therapeutic payload over time [22]. Chitosan-based NPs have a wide range of uses in non-parenteral drug administration, including the treatment of cancer, gastrointestinal illnesses, lung diseases, drug delivery to the brain, and ocular infections, as will be demonstrated. The prepared chitosan–AgNPs and chitosan–AuNPs were characterized using XRD, FTIR, TEM, and SEM, and were further tested for their antibacterial and antibiofilm properties.

### 3.6. Characterization of Chitosan–AgNPs and Chitosan–AuNPs

**Figure 4:**
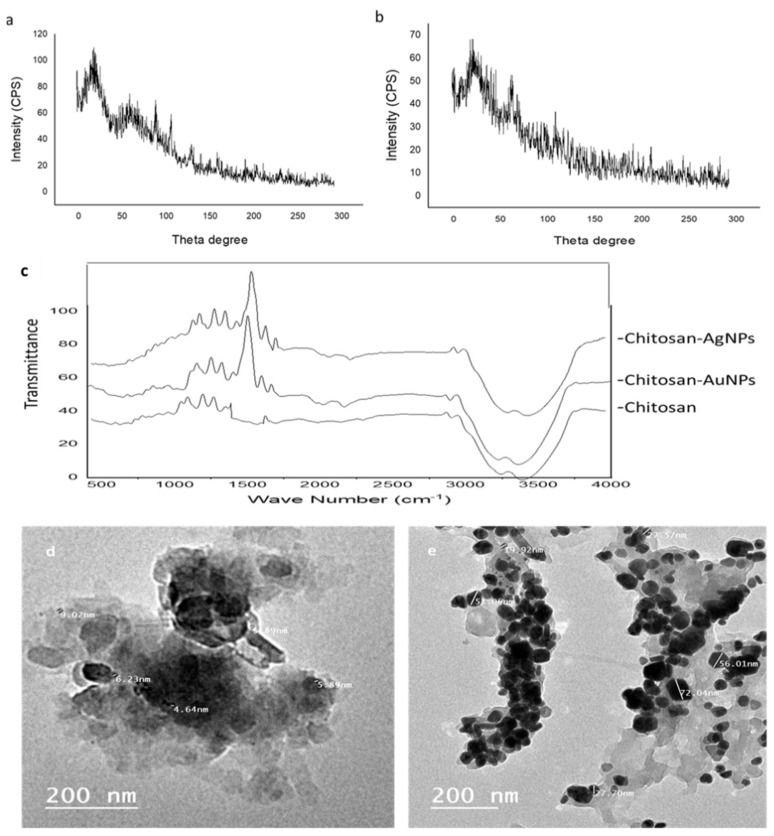
XRD of (**a**) chitosan-AgNPs, (**b**) chitosan-AuNPs, and (**c**) FTIR of chitosan, chitosan–AgNPs and chitosan–AuNPs; (**d**,**e**) TEM micrographs of chitosan–AgNPs and chitosan–AuNPs, respectively.

The XRD patterns of the chitosan–AgNPs and chitosan–AuNPs are shown in Figure 3. The XRD pattern of the chitosan–AgNPs exhibited a strong characteristic peak for chitosan at about 20° (Figure 4a), while the XRD showed another peak at about 64° and 77° corresponding to Ag nanoparticles in addition to the chitosan at 20° [23,24,25]. Additionally, the XRD pattern for chitosan–AuNPs showed a characteristic peak at about 20°, and for gold nanoparticles at 38°, and 66°. Figure 4 illustrates the FTIR spectra of chitosan, chitosan–AgNPs, and chitosan–AuNP conjugates synthesized with *Aspergillus* sp. AgNPs and *Alternaria* sp. AuNPs. The peaks that appear at 3000–3700 cm^−^^1^ are the characteristic bands for NH2 and OH groups [26]. The carbonyl (C=O) stretch vibration was displayed at 1641.33 cm^−1^ in chitosan [27]. For the FTIR of chitosan–AuNPs and chitosan–AgNPs, new stretching vibration bands were observed near 1500 cm^−1^ in the Ag- and Au-loaded chitosan spectra. The appearance of this peak may result from the binding of Ag nanoparticles to the N–H bond of the chitosan [25,26,27,28]. The prepared chitosan–AgNPs and chitosan–AuNP conjugates were visualized using transmission electron microscopy (Figure 4).

### 3.7. Evaluation of Antibacterial Activity of AgNPs, AuNPs, and Chitosan–AgNP and Chitosan–AuNP Conjugates

**Figure 5:**
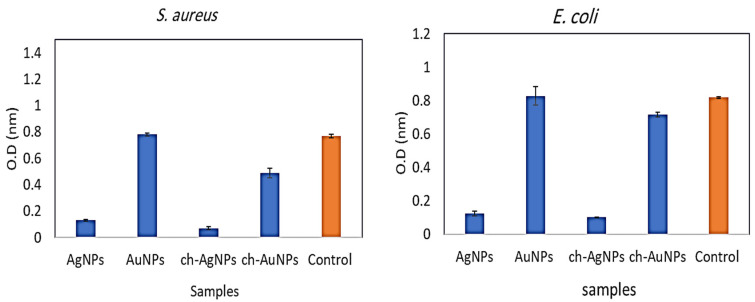
Antibacterial activity of the prepared chitosan–AgNPs and –AuNPs in comparison with biosynthesized nanoparticles.

Aspergillus AgNPs, Alternaria AuNPs, and chitosan–AgNP and chitosan–AuNP conjugates were investigated for their antibacterial properties against one Gram-positive bacterium, *S. aureus*, and one Gram-negative bacterium, *E. coli*. Figure 5 depicts the results. When applied alone, biosynthesized AgNPs demonstrated a bactericidal impact against *E. coli* and *S. aureus*. AuNPs, on the other hand, did not show antibacterial action against all pathogens examined. The chitosan–AgNP conjugation had robust antibacterial action against all microorganisms tested, whereas the antibacterial activity of chitosan–AuNPs was modest against *S. aureus*, but very low against *E. coli*. (Figure 5). Several reports have mentioned that silver nanoparticles have a stronger antimicrobial activity than gold nanoparticles; this may be due to a stronger plasmon resonance in the silver nanoparticle.

### 3.8. Antibiofilm Activity

The production of bacterial biofilms has been associated with the persistence of bacterial nosocomial infections. This method promotes bacterial colonization on living and non-living surfaces, and has been linked to 65–80% of all clinical illnesses. Because of these adaptive alterations, biofilm-forming bacteria are 10- to 1000-fold more resistant to traditional antibiotics, posing a significant challenge in developing antimicrobials specifically for biofilm treatment [22]. In this study, using a microtiter biofilm plate assay, the biosynthesized AgNPs, AuNPs, and chitosan–AuNPs, and chitosan–AgNPs were investigated for biofilm-inhibitory activity against four clinical biofilm-forming pathogenic bacterial clinical isolates from Egyptian hospitals. The findings revealed that biosynthesized AgNPs have significant antibiofilm efficacy against all bacteria that generate biofilms. On the other hand, the AuNPs, as expected, did not display any pronounced antibiofilm activity. The chitosan–AgNPs also displayed potent biofilm-inhibitory activity against all tested clinical isolates, while chitosan–AuNP conjugates showed moderate activity against *B. subtilis*, weak activity against *S. aureus*, and no antibiofilm action against the remainder of the microorganisms (Table 1).

## 4. Conclusions

From the obtained results, it was concluded that the utilization of fungal extracts for the biogenic synthesis of silver and gold nanoparticles is an eco-friendly, efficient, relatively inexpensive, and safe strategy. Moreover, the prepared chitosan–AgNPs and chitosan–AuNP conjugates displayed bioactive potentialities, including antibacterial activities against the tested microorganisms where chitosan–AgNPs showed better activity than the chitosan–AuNP. Anti-biofilm activity, against some pathogenic species showed also a much better results with chitosan–AgNPs which suggest the upper hand of using AgNP and this may due to a stronger plasmon resonance in the silver nanoparticle.

## Figures and Tables

**Table 1:** Biofilm-inhibitory activity of the prepared chitosan–AgNPs and –AuNPs in comparison with biosynthesized nanoparticles.

	Biofilm-Inhibition Ratio (%)			
	*E. coli*	*B. subtilis*	*S. aureus*	*P. aeruginosa*
AgNPs	86.79 ± 0.12	84.36 ± 0.17	80.54 ± 0.25	85.58 ± 0.19
AuNPs	0.00	29.43 ± 0.21	0.00	0.00
ch-AgNPs	87.39 ± 0.16	75.62 ± 0.10	89.88 ± 0.12	86.24 ± 0.14
ch-AuNPs	11.64 ± 0.17	50.62 ± 0.25	32.91 ± 0.21	3.57 ± 0.26
